# Recent Advances in Cell‐Based In Vitro Models to Recreate Human Intestinal Inflammation

**DOI:** 10.1002/advs.202301391

**Published:** 2023-09-21

**Authors:** Maria Helena Macedo, Mafalda Dias Neto, Lorenzo Pastrana, Catarina Gonçalves, Miguel Xavier

**Affiliations:** ^1^ INL – International Iberian Nanotechnology Laboratory Avenida Mestre José Veiga Braga 4715‐330 Portugal

**Keywords:** gut‐on‐chip, in vitro models, inflammatory bowel disease, intestinal inflammation, organoids, transwells

## Abstract

Inflammatory bowel disease causes a major burden to patients and healthcare systems, raising the need to develop effective therapies. Technological advances in cell culture, allied with ethical issues, have propelled in vitro models as essential tools to study disease aetiology, its progression, and possible therapies. Several cell‐based in vitro models of intestinal inflammation have been used, varying in their complexity and methodology to induce inflammation. Immortalized cell lines are extensively used due to their long‐term survival, in contrast to primary cultures that are short‐lived but patient‐specific. Recently, organoids and organ‐chips have demonstrated great potential by being physiologically more relevant. This review aims to shed light on the intricate nature of intestinal inflammation and cover recent works that report cell‐based in vitro models of human intestinal inflammation, encompassing diverse approaches and outcomes.

## Graphical abstract

The ever‐growing plethora of cell‐based models that recapitulate intestinal inflammation has experienced significant improvements in physiological relevance with accompanying complexity. While all models are by definition wrong it becomes crucial to choose wisely the model that better fits the biological question at hand. This review summarizes the latest updates in the field aiming to aid the reader in this quest.

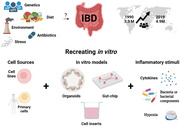

## 1 Introduction

Inflammatory bowel disease (IBD) is a gastrointestinal chronic disease that comprises myriad conditions, with Crohn's disease and ulcerative colitis representing the main subtypes.^[^ ^1^ ^]^ IBD causes life‐impairing symptoms – abdominal pain, diarrhea, bloody stools, and weight loss – and patients are more prone to develop colorectal cancer.^[^ ^2^, ^3^, ^4^ ^]^ There were 3.3 million estimated cases of IBD in 1990 and, by 2019, the number had risen to 4.9 million cases worldwide. The prevalence of IBD is estimated to be higher in the US and China.^[^ ^5^ ^]^ Since IBD is normally diagnosed at a young age, global health care costs will keep increasing.^[^ ^3^ ^]^

The exact causes of IBD and its progression are not entirely understood but different factors appear to be involved, including uncontrolled immune responses in genetically prone individuals, diet, and environmental factors.^[^ ^6^, ^7^, ^8^, ^9^ ^]^ Due to its unknown pathogenesis, IBD therapy focuses mainly on tackling and controlling the inflammatory processes. Anti‐TNF agents emerged as a revolutionary solution for the standard use of immunosuppressants and immunomodulators, enabling long‐term remission of the disease.^[^ ^10^ ^]^ However, primary nonresponse, loss of response, and adverse effects are still observed, demanding novel approaches and treatment alternatives, whose efficacy has to be tested prior to being used in clinical practice.^[^ ^11^ ^]^

**Figure 1:**
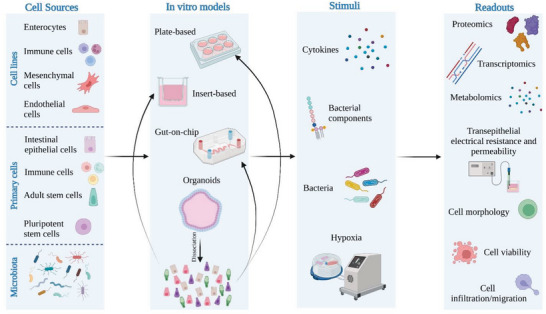
Variables in in vitro models of intestinal inflammation. Models can be obtained using different technologies and comprehend different cell types, including or not microbiota. Different stimuli can be applied to induce inflammation, and multiple outputs can be measured. Created with BioRender.com.

Being able to replicate intestinal inflammation in vitro can help unravel key mechanisms that govern the onset of the disease. Thus, In vitro models, ever more complex and realistic, are a promising tool to study IBD pathogenesis and treatment. These platforms can also be used as screening tools for novel anti‐inflammatory drugs or foods. However, there is a lack of standardized protocols to obtain in vitro models of intestinal inflammation. This is not due to scarce studies within the field. On the contrary, it is due to abundant literature that is typically inconsistent regarding critical parameters, including the number and type of different cells used, the inflammatory stimuli (type, concentration, site) applied, and even the measured outputs (**Figure** **1**).

This review aims to bring this into perspective, offering a complete overview of the different models that are used today, focusing on recent publications, mainly from the last quinquennium. It covers studies using both immortalized and primary cells on simpler multiwell plates and insert‐based models, to more complex intestinal organoids and organ‐on‐chips, which have arisen as key enabling technologies with significant potential impact on future research.

## 2 Intestinal Physiology and Inflammation

The small intestine is the longest part of the digestive system, where most absorption of nutrients, water, electrolytes, and xenobiotics occurs. It is formed by three different sections: (i) the duodenum, leading from the stomach and with connections to the liver and pancreas; (ii) the jejunum and (iii) the ileum, the distal and longest section.^[^ ^12^ ^]^ The wall of the small intestine is divided into four layers: mucosa, submucosa, muscularis, and serosa. The innermost layer is the mucosa, subdivided into the mucous epithelium, the lamina propria, and the muscularis mucosae.^[^ ^13^ ^]^ The presence of finger‐like protrusions – villi – and brush border microvilli enhance the intestinal surface area and facilitate the contact between cells and the luminal contents, promoting absorption. The minute depressions of the mucosa that extend into the lamina propria – crypts – are interspaced between the villi. The intestinal epithelium provides a physical and (bio)chemical barrier, functioning as a dynamic interface between the body and the external environment.^[^ ^14^, ^15^ ^]^ The epithelium is formed by a single layer of different intestinal epithelial cells (IECs), including enterocytes, goblet cells, enteroendocrine cells, and Paneth cells, which are connected by tight and adherens junctions (TJs and AJs) that form the apical junction complex and regulate paracellular transport. IECs derive from the intestinal stem cells that reside at the crypts base.^[^ ^16^ ^]^ Cellular self‐renewal occurs every 3–4 d and maintains the secretory and absorptive functions of the epithelium, and preserves its continuous mucous barrier.^[^ ^17^ ^]^ The lamina propria lies underneath the epithelium and comprises immune cells, fibroblasts, and stromal stem cells, among other cell types.^[^ ^18^, ^19^, ^20^, ^21^ ^]^ The epithelial‐mesenchymal crosstalk is mainly driven by the release of soluble factors – growth factors, cytokines, and enzymes – and shapes crucial functions of the small intestine including cell differentiation and polarization. The communication between cells also contributes to cellular turnover and promotes the establishment of the intestinal stem cell niche, contributing to maintaining mucosal integrity.^[^ ^22^ ^]^

**Figure 2:**
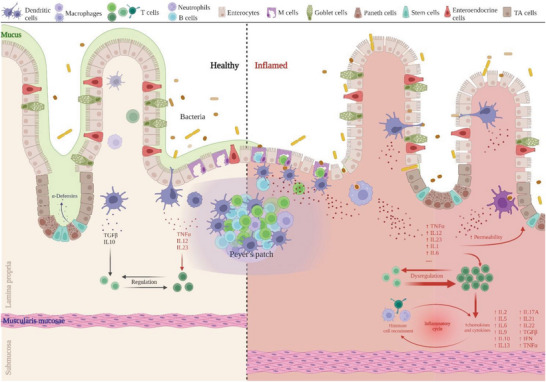
Healthy versus inflamed intestinal mucosa. In the healthy state, the epithelial barrier is intact and a continuous layer of mucus covers the epithelium. A balance of pro and anti‐inflammatory cytokines from non‐activated immune cells leads to overall intestinal homeostasis. In the inflamed state, tight junctions are disrupted and the mucous layer is reduced or absent, leading to an increased permeability and luminal antigen uptake. DCs and macrophages become activated and can recognize nonpathogenic bacteria (commensal microbiota), releasing pro‐inflammatory cytokines and exacerbating the inflammatory response, causing further barrier disruption. (TA – Transit‐amplifying; TFG – Transforming Growth Factor; IL – Interleukin; TNF – Tumor Necrosis Factor; IFN – Interferon). Adapted from “Intestinal Immune System (Small Intestine)”, by BioRender.com (2022). Retrieved from https://app.biorender.com/biorender‐templates.

During an inflammatory state, the crosstalk between cells is disrupted, altering cellular pathways, and contributing to the development and perpetuation of inflammation.^[^ ^22^ ^]^ The inflamed intestine is characterized by a loss of barrier integrity, caused by disturbances in TJ proteins, but it remains unclear whether this is a cause or a consequence of inflammation.^[^ ^15^ ^]^ Barrier dysfunction enables a closer interaction between the luminal contents and the immune cells of the lamina propria, leading to the infiltration of pro‐inflammatory cells, and consequent release of pro‐inflammatory cytokines and reactive oxygen/nitrogen species, contributing to a sustained activation of the mucosal immune system.^[^ ^21^, ^23^, ^24^, ^25^ ^]^ **Figure** **2** shows a representation of the main changes occurring in an inflamed intestinal epithelium. These can be related to the histological sections from human biopsies and the inflamed models obtained in vitro shown in Figure 4A,B.

The gut microbiota also play a key role in maintaining gut homeostasis, influencing metabolism, immunity, and barrier function. Commensal microbes promote host resistance against infectious agents by competing for space and/or nutrients directly with pathogens, and by mediating protective host immune responses.^[^ ^26^, ^27^ ^]^ When there is an imbalance in the composition of the microbiota – dysbiosis – it can lead to an inflammatory state.^[^ ^28^ ^]^ Recent studies show that some enteric pathogens can outcompete commensal microbes by triggering inflammation, favoring pathogen invasion and dissemination.^[^ ^23^, ^24^, ^25^ ^]^ Antibiotics prescribed to treat bacterial infections can have unintended consequences on the gut microbiota by reducing diversity and altering the composition of beneficial bacteria, in a way that promotes inflammation. In addition to antibiotics, environmental stressors, such as exposure to pollutants, toxins, and other environmental agents, can also disrupt the balance of the gut microbiota leading to inflammation. Furthermore, insufficient sleep can have an impact on the composition and diversity of the gut microbiota. Moreover, inadequate sleep can increase the presence of inflammatory markers, contributing to a dysregulation of the immune system, and contributing to inflammation.^[^ ^28^ ^]^

Psychological stress has also been found to have an impact in the gut as it can lead to an increased release of pro‐inflammatory effectors.^[^ ^29^ ^]^ In situations of acute stress, the secretion of the pro‐inflammatory cytokines interleukin (IL)‐6, TNF‐α, and IL‐1β is increased, with stress inducing long‐lasting harmful consequences to the health of the intestinal barrier.^[^ ^30^ ^]^

Engaging in physical activity can help regulate stress levels and improve sleep quality, indirectly contributing to gut health. Additionally, physical activity has positive effects on inflammation. Regular exercise supports the growth of beneficial bacteria and increases microbial diversity. It also helps decreasing the production of pro‐inflammatory molecules, promotes the release of anti‐inflammatory compounds, and improves barrier function.^[^ ^28^ ^]^

### 2.1 The Role of Cytokines

Cytokines are implicated in the pathogenesis of IBD.^[^ ^27^ ^]^ Several cytokines control IEC activation, survival, migration, and differentiation, as well as the secretion of antimicrobial peptides, having a fundamental role in mucosal healing.^[^ ^27^ ^]^ An imbalance between pro‐ and anti‐inflammatory cytokines leads to disease perpetuation and tissue destruction, highlighting the significance of cytokines as key players in the inflammatory process. For example, interferon (IFN)‐γ and tumor necrosis factor (TNF) can act on TJs and induce IEC apoptosis, leading to loss of barrier function and aggravating inflammation.^[^ ^23^, ^26^, ^27^ ^]^

Dendritic cells (DCs) and macrophages, present in the intestinal lamina propria, are key antigen‐presenting cells (APCs) and can secrete large amounts of pro and anti‐inflammatory cytokines.^[^ ^31^ ^]^ Nonetheless, various nonimmune cells such as epithelial cells, subepithelial myofibroblasts, adipocytes, and stromal fibroblasts, can also secrete cytokines and activate mucosal immune cells, contributing to inflammation.^[^ ^27^, ^32^, ^33^, ^34^ ^]^ For example, TNF has been detected in great amounts in patients with IBD, being produced by CD14^+^ macrophages, adipocytes, fibroblasts, and T cells.^[^ ^27^, ^35^, ^36^ ^]^

Other cytokines have been detected in patients with IBD. Some examples include IL‐6, which can activate APCs and T‐cells;^[^ ^37^ ^]^ IL‐10 and transforming growth factor (TGF)‐β, which are produced by regulatory T (T_Reg_) cells to control inflammation;^[^ ^38^, ^39^ ^]^ and the IL‐12 and IFN families, which are produced by APCs and can have a pro‐ or anti‐inflammatory role.^[^ ^27^, ^40^ ^]^

It is generally accepted that some cytokines have a pro‐inflammatory effect, such as IL‐1β/α, IL‐6, IL‐17, IL‐18, IL‐33, IFN‐γ, and TNF‐α, while others have an anti‐inflammatory effect, as it is the case of IL‐4, IL‐10, and IL‐13. It is important to stress however that cytokines can have different biological activities and exert pro‐ or anti‐inflammatory effects depending on their local concentration.^[^ ^41^ ^]^ The traditional pro‐ and anti‐inflammatory labels are too simplistic and can be deceiving when used to describe the actions of cytokines. For example, although generally considered as pro‐inflammatory cytokines contributing to the pathology of IBD, some studies show that TNF, IL‐6, and IL‐17 promote epithelial proliferation.^[^ ^42^, ^43^, ^44^, ^45^, ^46^ ^]^

## 3 Cellular Biology of the Gut

### 3.1 Cell Types

#### 3.1.1 Epithelial Cells

Enterocytes are the main cell population of the human small intestine epithelium, making up for more than 80% of all epithelial cells. Besides their metabolic, absorptive, and barrier functions, enterocytes are considered accessory immune cells expressing cytokines and other proteins associated with immune functions, such as the polymeric immunoglobulin receptor, which is associated with immunoglobulin A secretion, major histocompatibility complex class II molecules involved in antigen presentation, and enzymes, such as cyclooxygenase II and 12‐lipoxygenase, involved in the synthesis of lipid mediators of inflammation.^[^ ^47^, ^48^, ^49^ ^]^

Goblet cells are specialized cells that secrete mucins to form a thick mucus layer, which acts as a barrier and limits interactions with luminal contents. The mucous layer is highly dynamic, and compositional alterations influence many pathological conditions, including IBD.^[^ ^50^ ^]^ Goblet cells are involved in innate immunity by secreting antimicrobial proteins, cytokines, and chemokines.^[^ ^51^ ^]^ A recent study showed that goblet cells can uptake luminal antigens and present them to underlying APCs, eliciting adaptive immune responses.^[^ ^52^ ^]^

Paneth cells are secretory cells that reside at the base of intestinal crypts. They secrete antimicrobial, immune system‐stimulating, and trophic molecules, as well as cytokines, such as IL‐17A and TNF‐α. Paneth cells signal intestinal stem cells contributing to the proliferation and differentiation of the intestinal epithelium.^[^ ^53^ ^]^ Besides, Paneth cells respond to inflammation and contribute to tissue regeneration by acquiring stem‐like properties.^[^ ^54^ ^]^

Enteroendocrine cells are also secretory cells and are scattered throughout the small intestine, comprising less than 1% of epithelial cells. They produce hormones that can act locally or systemically to regulate various functions in the gastrointestinal tract – such as food intake, digestion, and motility. Enteroendocrine cells are key sensors of microbial metabolites, releasing cytokines in response to pathogen‐associated molecules and have been pointed out as orchestrators of intestinal inflammation.^[^ ^55^ ^]^

In vivo, M‐cells are located in the FAE and have an exceptional capacity to uptake microbes, antigens, and other particles, and present them to the underlying immune cells to inhibit or trigger immune responses.^[^ ^56^, ^57^ ^]^ Owing to these characteristics, M‐cells can increase intestinal permeability, and play a crucial role in food and drug tolerance.^[^ ^58^, ^59^, ^60^ ^]^

#### 3.1.2 Immune Cells

Immune cells can be grouped into two categories: myeloid cells, including granulocytes, monocytes, macrophages, and DCs; and lymphoid cells, such as B, T, and natural killer cells, which act together mediating intestinal inflammation.^[^ ^61^, ^62^ ^]^

Granulocytes – including eosinophils, basophils, neutrophils, and mast cells – maintain intestinal homeostasis by (i) eliminating invading microbes, (ii) releasing soluble factors, such as histamine and prostaglandins, and (iii) increasing tissue permeability thus allowing phagocyte migration.^[^ ^63^, ^64^, ^65^ ^]^ However, their excessive recruitment under pathological conditions such as IBD is associated with mucosal injury.^[^ ^66^ ^]^

Macrophages are APCs and secrete different cytokines, such as TNF‐α, IL‐1β, IL‐6, IL‐8, and IL‐23.^[^ ^67^ ^]^ Given their phagocytic capacity, macrophages are involved in microbial clearance.^[^ ^62^ ^]^ DCs, another type of APCs, are among the first immune cells to come into contact with foreign particles and are pivotal in shaping the immune response.^[^ ^62^ ^]^ They present antigens to naïve T cells, driving their activation, proliferation, and differentiation.^[^ ^68^ ^]^

T lymphocytes migrate from peripheral lymphoid organs to the gut‐associated lymphoid tissues (GALT) in the lamina propria or to the follicle‐associated epithelium (FAE), where they are coined intraepithelial lymphocytes.^[^ ^69^ ^]^ T cells prevent pathogenic invasion and regulate immune responses and tissue damage.^[^ ^69^ ^]^ B cells can also be present both in the GALT and in the FAE,^[^ ^70^ ^]^ and intervene in the immune response by producing immunoglobulins and immune‐modulatory cytokines, such as IL‐10.^[^ ^71^ ^]^ Natural killer (NK) cells are also present in the GALT and are involved in mucosal innate immunity due to their cytolytic activity.^[^ ^72^ ^]^

### 3.2 Cell Sources

#### 3.2.1 Cell Lines

Cell lines are readily available, cost‐effective, easy to use, and bypass many ethical concerns. Their reproducibility and virtually unlimited lifespan are also frequently pointed as main strengths. However, these also raise some concerns, as being genetically manipulated, they become susceptible to alterations in their phenotype, function, and response to stimuli, all of which can be aggravated by serial passaging, leading to intra‐ and interlaboratory variability. Regarding intestinal inflammation, the lack of cell lines representing the cellular diversity implied in this condition hinders the recreation of a comprehensive cell‐based in vitro model.^[^ ^73^ ^]^

Caco‐2 and HT29‐MTX mimic enterocytes and goblet cells respectively, and are the most commonly used cell lines in intestinal in vitro models. The T84 cell line, derived from a metastatic human colon carcinoma, can proliferate and spontaneously differentiate into an absorptive epithelial monolayer. But, unlike Caco‐2, T84 retain their original colonic features upon differentiation.^[^ ^74^ ^]^ HIEC‐6 is a human intestinal epithelial cell line of embryonic origin that is unable to maintain an elevated trans‐epithelial electrical resistance (TEER) over time and typically develops into an interrupted cell monolayer, suggesting an immature epithelium.^[^ ^75^ ^]^ NCI‐H716, a cecal adenocarcinoma‐derived cell line, has been used as surrogate enteroendocrine cells. However, NCI‐H716 fails to express relevant hormones, such as cholecystokinin and peptide YY, and their main use has been in studies of GLP‐1 regulation.^[^ ^76^, ^77^ ^]^ Regarding Paneth cells, there is a lack of immortalized cell lines and much of the knowledge about their biology has been generated using animal models or primary cells. For example, enteroid cultures have been used to evaluate interactions between Paneth cells and the intestinal microbiota.^[^ ^78^ ^]^

A variety of cell lines have been used as surrogate immune cells in intestinal in vitro models. THP‐1, MUTZ‐3, and U937 are human monocytic leukemia cell lines.^[^ ^79^, ^80^, ^81^, ^82^ ^]^ Both U937 and THP‐1 can be differentiated into macrophages,^[^ ^83^, ^84^ ^]^ while MUTZ‐3 are used to obtain DCs.^[^ ^80^ ^]^ THP‐1 is, undoubtedly, the most used cell line to surrogate immune cells in in vitro models.

Raji B is a B lymphocyte cell line that is used to promote the differentiation of Caco‐2 into M‐like cells. Given the key role that M cells can play in intestinal inflammation, Raji B will be addressed in more detail in Section 3.2.1.4.^[^ ^85^, ^86^ ^]^

##### Caco‐2

Caco‐2 originate from a human colorectal carcinoma. When cultured in vitro, Caco‐2s develop a polarized monolayer with distinct apical and basolateral plasma membrane domains and well‐established TJs. Caco‐2 cells are responsive to cytokines, such as IL‐1β, TNF‐α, and INF‐γ.^[^ ^15^ ^]^ TNF‐α, for example, can increase the permeability of Caco‐2 monolayers by activating the nuclear factor kappa‐light‐chain‐enhancer of activated B cells (NF‐κB), leading to a downregulation of ZO‐1.^[^ ^87^ ^]^ Similar downregulation can be observed when there is an increase in myosin light‐chain kinase (MLCK) 1.^[^ ^88^ ^]^

##### HT29‐MTX

HT29‐MTX secrete growth factors (platelet derived growth factor AA, TGF‐α, and TGF‐β), pro‐inflammatory cytokines (TNF‐α, IL‐1β, and IL‐6), immune‐modulatory cytokines (IL‐3 and G‐ and GM‐granulocyte colony‐stimulating factor), and chemokines (fractalkine, IL‐8, monocyte chemoattractant protein‐1, and interferon gamma‐induced protein 10) in similar levels to those found in vivo.^[^ ^89^ ^]^ HT29‐MTX have been used ubiquitously in coculture with Caco‐2 supplementing the lack of mucus production of the latter and decreasing overall barrier tightness.^[^ ^90^ ^]^

##### THP‐1

THP‐1 cells can be differentiated into macrophages using phorbol 12‐myristate 13‐acetate (PMA) but there is a lack of consistency regarding the conditions of the PMA stimulus.^[^ ^91^ ^]^ Concentrations range from 10 to 60 ng mL^−1^ and durations from 4 to 65 h, leading to discrepant outcomes.^[^ ^92^, ^93^, ^94^, ^95^, ^96^, ^97^, ^98^, ^99^, ^100^, ^101^ ^]^ When THP‐1 are exposed to PMA for longer and/or at higher concentrations, they will likely elicit a stronger immune response. Marescotti and colleagues^[^ ^98^ ^]^ observed a dose‐dependent increase in the secretion of IL‐8 and TNF‐α by THP‐1s. Establishing standard conditions will be critical to avoid inter‐laboratory variability and achieve reproducible cellular function.

##### Raji B

Raji B promote the differentiation of Caco‐2 into M‐like cells.^[^ ^102^, ^103^ ^]^ Kernéis et al.^[^ ^104^ ^]^ were the first to establish an M cell model by culturing Caco‐2 cells on the basolateral side of cell culture inserts and Raji B on the apical side, resulting in an epithelial monolayer with embedded lymphocytes and M‐like cells. Gullberg and colleagues^[^ ^105^ ^]^ inverted this approach and seeded Caco‐2 on the apical side and Raji B on the basolateral culture well. Given that Raji B grow in suspension, this showed that the soluble factors released by Raji B were sufficient to promote the conversion of Caco‐2 into M‐like cells. Des Rieux et al.^[^ ^106^ ^]^ improved the conversion of M cells by 15–30% by seeding Caco‐2 cells on the apical side of the inserts and then inverting the inserts to seed Raji B cells on a reservoir created ad‐hoc.^[^ ^103^ ^]^ Other variables, such as seeding densities, pore size, frequency of culture medium changes, and the use of coatings, have led to variable results.

To the best of our knowledge, there are no examples of in vitro models mimicking intestinal inflammation that include M cells. However, we believe their use should be considered given the critical role they play in the gut immunity.^[^ ^107^ ^]^

#### 3.2.2 Primary Cells

Human primary cells offer increased physiological relevance, and higher in vitro to in vivo correlation when compared to cell lines. They also open up avenues for the development of therapies adapted to patient‐specific needs. While this could at first seem cost‐prohibitive, there is high potential for their early adoption in studies targeting genetic subpopulations, gender differences, or subgroups with specific disease comorbidities.^[^ ^108^ ^]^

Primary cells are harvested from tissue samples and can be used directly or further grown in vitro. Stem‐cell based strategies are normally used for the latter approach surmounting the limited lifespan associated with fully‐differentiated cells. In this section we will focus on the two main stem cell classes typically used to derive intestinal epithelial cells, adult (ASCs) and pluripotent (PSCs) stem cells, and will discuss different strategies to obtain primary immune cells.

##### Adult Stem Cells

Intestinal ASCs are epithelial cells resident at the base of intestinal crypts, which can retain various features from their site of origin as well as any genetic or epigenetic alterations. This is particularly pertinent for models using IBD patient‐derived cells, which can potentially mimic disease‐specific environments in vitro. These cells can be isolated from surgical resections or biopsy samples, and further expanded as whole intestinal crypts or as single stem cells. Since conventional 2D cultures typically result in rapid loss of stemness, most studies use ASCs to derive intestinal organoids with complex 3D cellular organization (further detailed in Section 4.2. of this review). Nevertheless, Wang and co‐workers were able to successfully establish a planar, self‐renewing monolayer of intestinal crypts cultured on a collagen hydrogel.^[^ ^109^, ^110^, ^111^ ^]^ Additionally, they demonstrated that when exposed to TNF‐α and INF‐γ, stem cell proliferation was suppressed due to diminished self‐renewal capability rather than increased cell death.^[^ ^110^ ^]^

Stem cells have the ability to generate the most representative epithelial cells residing in the intestinal tissue, including M cells.^[^ ^112^, ^113^ ^]^ Contrasting with strategies using Caco‐2 cells, inducing an M‐like phenotype in primary cells is simpler and fairly invariable. The addition of receptor activator of NF‐κB ligand (RANKL) to both mouse and human cultures induced M cell‐associated gene expression along with enhanced uptake of microparticles and bacteria.^[^ ^112^, ^114^ ^]^ Interestingly, Wood and collaborators demonstrated that TNF‐α works synergistically with RANKL to further boost the expression of M cell specific genes.^[^ ^115^ ^]^

MatTek sells 3D human tissue models obtained from primary human intestinal stem cells from a single donor and grown at an air–liquid interface (EpiIntestinal).^[^ ^116^, ^117^ ^]^ The model incorporates different epithelial cells – enterocytes, Paneth cells, M cells, tuft cells, and stem cells – in a polarized epithelium including functional tight junctions and brush borders, and has been used in intestinal inflammation studies following exposure to pro‐inflammatory cytokines.^[^ ^116^, ^118^, ^119^, ^120^ ^]^

##### Pluripotent Stem Cells

Pluripotent stem cells (PSCs) can be of embryonic origin or be attained by reprogramming somatic cells.^[^ ^121^ ^]^ Contrary to adult stem cells, PSCs can be used to derive both stromal and immune cells.^[^ ^122^ ^]^ Organoids derived from PSCs have the ability to spontaneously generate mesenchyme in addition to epithelial cells, enabling more complex investigations.^[^ ^123^, ^124^ ^]^

PSC‐derived cells can generate tissue‐engineered small intestine with appropriate cellular identities and contractility.^[^ ^125^ ^]^ Kandilogiannakis et al.^[^ ^123^ ^]^ developed a human intestinal organoid model from a human embryonic stem cell line. The organoids were kept up to 13 passages with stable structure, including intestinal stem cell niches, enterocytes, goblet cells, and enteroendocrine cells. Naumovska et al.^[^ ^126^ ^]^ and Workman et al.^[^ ^127^ ^]^ used iPSCs to obtain functional intestinal epithelial tissue in organ‐chip devices. While the former seeded and differentiated iPSCs directly on‐chip, the latter followed a multistep methodology where cells were first cultured off‐chip to form definitive endoderm, epithelial structures, and organoids, which were later dissociated into single cells, FACS‐enriched for CD326 expression and finally cultured on‐chip.

Despite being a powerful tool for precision medicine, PSC technology still faces serious technical constraints due to the poorly standardized differentiation protocols that are very complex and involve multiple highly specific steps.^[^ ^128^ ^]^ This leads to heterogeneity among cultures and results in a premature phenotype, displaying more embryonic and fetal features than those exhibited by adult stem cell‐derived models (de Souza, 2018). The long time needed to obtain patient‐derived PSCs is also a downside and may prevent the translation of such approaches to the clinic.^[^ ^129^ ^]^

##### Primary Immune Cells

Many intestinal in vitro models incorporate primary immune cell subpopulations, including lymphocytes,^[^ ^130^, ^131^, ^132^, ^133^, ^134^ ^]^ monocytes/macrophages,^[^ ^135^, ^136^ ^]^ dendritic cells,^[^ ^137^ ^]^ and neutrophils.^[^ ^138^, ^139^ ^]^

Tissue‐resident immune cells can be harvested from the intestinal lamina propria and show tissue‐specific characteristics and functions, being phenotypically different from the ones found in other tissues or in circulation and are thus more physiologically relevant.^[^ ^67^, ^132^, ^137^ ^]^ Although scarce, resected tissue pieces remain the preferred option since biopsies often fail to provide sufficient cell numbers.^[^ ^131^, ^132^, ^134^, ^137^ ^]^ Obtaining immune cells from whole blood is easier as these are enriched from peripheral blood mononuclear cell (PBMC) populations.

In culture, these cells have limited lifespan and expansion capacity.^[^ ^140^ ^]^ Thus, cryopreservation is recommended until epithelial cultures and models are established.^[^ ^128^ ^]^ iPSCs have also been directed towards innate immune cell differentiation, particularly into macrophages and lymphocytes.^[^ ^122^, ^130^, ^134^, ^135^, ^136^, ^139^, ^141^ ^]^ However, their use towards modelling intestinal inflammation has been only occasional.

### 3.3 Intestinal Microbiota

The gut microbiota plays a crucial role in human health, with recent estimates indicating that the number of bacteria in humans actually exceeds the number of human cells.^[^ ^142^ ^]^ The microbiota provides essential nutrients to the host, defends against pathogen colonization, and regulates the immune system.^[^ ^143^ ^]^ Everyone's microbiota is unique depending on age, geographical location, environment, use of medication, and diet.^[^ ^144^ ^]^ Nevertheless, Proteobacteria, Firmicutes, Actinobacteria, and Bacteroidetes are the most prevalent phyla found in the gut.^[^ ^145^ ^]^

Upon colonization, the naïve mucosa is primed to launch transient inflammatory cascades, which are essential to establish a homeostatic coexistence. For example, commensal bacteria metabolize indigestible carbohydrates forming short‐chain fatty acids (SCFAs), which reduce local inflammation, protect against pathogen infiltration, and maintain barrier integrity by regulating host immune cells.^[^ ^146^ ^]^ When IECs are stimulated by butyrate (a type of SCFA), they synthesize PPAR‐γ, which decreases nitric oxide synthase (NOS) and prevents the creation of inducible NOS (iNOS) and nitrate, which are energy sources for pathogens.^[^ ^147^ ^]^ PPAR‐γ also contributes to maintaining a hypoxic environment, suppressing the growth of facultative anaerobic pathogens, such as Enterobacteriaceae, which are associated with gut dysbiosis.^[^ ^148^, ^149^ ^]^ The microbiota also interacts with the host mucosal immune system through the GALT and helps “educating” immune cells to distinguish friends from foes.^[^ ^150^ ^]^

Breaking the balance between the host and the gut microbiota can lead to invasion and excessive reproduction of opportunistic pathogens that secrete pro‐inflammatory cytokines and activate pro‐inflammatory cascades, triggering the innate and adaptive immune system. Specialized immune cells are recruited and can ultimately skew the microbiota composition to increased ratios of pathobionts.^[^ ^143^ ^]^ These reciprocal interactions between the intestinal microbiota and immune system contribute to the pathogenesis of IBD.^[^ ^151^ ^]^ Patients with IBD show significant differences in microbial flora composition compared to healthy individuals: a decrease in obligate anaerobic bacteria diversity, commonly found in normal physiological conditions, paired with an overgrowth of facultative anaerobic species.^[^ ^152^ ^]^ Thus, it is clear that gut microbiota should be considered in in vitro models of intestinal inflammation.

## 4 Mimicking Inflammation In Vitro

In vitro models have been used for many decades and continuous efforts have advanced the field, contributing to bridge the gap between benchtop protocols and in vivo testing. While animal models provide critical insights into disease mechanisms and are unlikely to be replaced, they frequently fail to predict the results obtained in human clinical trials.^[^ ^108^, ^153^ ^]^ Distinct cellular and molecular mechanisms, as well as microbiota composition, leads to interspecies variability and make animal models unable of recapitulating the full spectrum of manifestations observed in humans.^[^ ^154^ ^]^ For example, chemically induced animal models of colitis can recapitulate the events of acute mucosal injury but differ from human IBD and inadequately address the chronic phase of gut inflammation.^[^ ^155^ ^]^

The use of simple in vitro models has allowed studying the interaction between different cytokines and their role in the activation of signaling pathways involved in inflammation.^[^ ^100^, ^156^ ^–^ ^166^ ^]^ With time, in vitro models are becoming more sophisticated and capable of mimicking organ physiology and disease. The intricacy and in vivo resemblance attained with the incorporation of nonepithelial components, such as stroma, immune cells, and microbiota, allows a detailed understanding of intestinal interactions. This helps to uncover the mechanisms of action behind certain diseases, and contributes to identify novel therapeutic targets and routes.^[^ ^128^, ^167^ ^]^

However, while it is true that in vitro models are becoming more representative of the human intestine, there will always be limitations. In vitro models inherently present a simplified representation of the complete organ, as fully replicating the intricate complexity and heterogeneity of the human intestine proves extremely challenging.^[^ ^168^, ^169^ ^]^

When considering inflammation, existing models often tend to focus on specific aspects, which can introduce limitations in capturing the complete pathophysiological complexity of the condition. This hinders the ability to accurately represent the full inflammatory response and makes it challenging to fully comprehend the underlying mechanisms involved. Besides, there is an oversimplification of the interactions that exist in vivo, such as cell–cell communication, immune cell migration, and the dynamic interplay between different components of the immune system. This oversimplification, along with a neglect of the interactions with cytokines and other signaling molecules, can lead to an incomplete understanding of the immune response.^[^ ^170^ ^]^ The lack of a systemic response is another disadvantage of in vitro models. Since the models are usually isolated systems, except for chips that can mimic different organs, they do not account for systemic responses observed in vivo.

Given the differences found when compared to the in vivo scenario, in vitro models may have a limited predictive power to assess the effectiveness of anti‐inflammatory compounds, challenging the translation of laboratory findings to clinical applications.^[^ ^171^ ^]^ The integration of patient specific cells, by using organoids, can be a way of counteracting this disadvantage).^[^ ^108^ ^]^ With personalized models, researchers can study IBD in specific individuals or subpopulations, and develop targeted therapies.^[^ ^172^ ^]^

High‐throughput screening is another advantage of in vitro models. Conventional well‐plates and insert‐based models can be used simultaneously, while organ‐chips can be automated and parallelized aiming for increased throughput.^[^ ^173^, ^174^ ^]^ Cost is another plus of in vitro models: a recent survey estimated that pharmaceutical companies can save up to 25% by using microphysiological systems like organ‐chips.^[^ ^175^ ^]^ Besides, in vitro models bypass some ethical concerns, being in line with the 3Rs principles of replacement, reduction, and refinement.^[^ ^176^ ^]^ However, it is important to state that ethical considerations still arise regarding the use of human‐derived cells or tissues and it is paramount to ensure proper consent and ethical sourcing of cells.^[^ ^169^ ^]^

**Figure 3:**
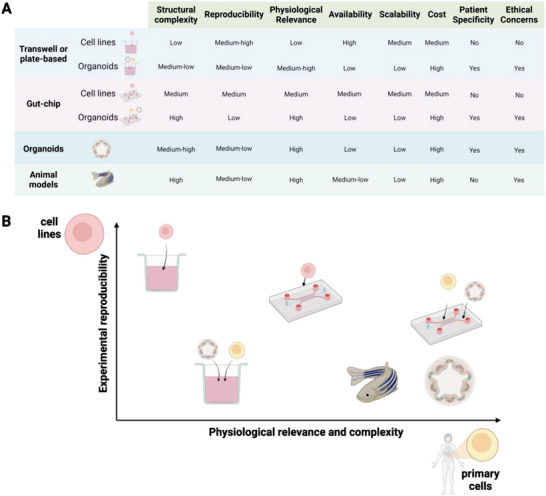
Comparative Evaluation of Models of Human Intestinal Inflammation – In the words of George E.P. Box, “All models are wrong, but some are useful.” When it comes to addressing biological questions, selecting the appropriate model ultimately requires striking a balance between various factors, including physiological relevance and experimental reproducibility. A and B present a qualitative comparison of the different models to assist readers in promptly identifying their primary strengths and weaknesses. Created with BioRender.com.

**Figure** **3** provides a comprehensive overview of different models employed in the study of intestinal inflammation, including animal models. Figure 3A compares the performance of these models across various key parameters, highlighting their respective strengths and limitations, while Figure 3B offers a visual representation of how they compare regarding the most important features when considering which model to adopt in the lab. By examining the advantages and pitfalls of each model type, researchers can make informed decisions regarding the selection of an appropriate model that aligns with their specific research objectives.

## 5 In Vitro Models of Gut Inflammation

Technological advances in cell culture have sprouted over the last few years, leading to a sharp evolution in the field of in vitro models. Allied with extended research in intestinal disorders, this has led to the development of myriad in vitro models that aim to mimic the intestinal tissue, either in health or disease, with the goal of replacing or at least minimizing in vivo experiments.^[^ ^15^ ^]^ In vitro models are developed according to the needs of specific research questions and goals but standardization is crucial to allow the harmonization of protocols and comparison of experimental outcomes. In the following chapters we will cover the latest advances in the field, which are summarized in **Table** **1**.

**Table 1:** In vitro models of human intestinal inflammation using well plates or cell culture inserts, gut‐on‐chip or organoids.

Model	Cell types		Microbiota	Stimuli^a)^	Readouts	Reference
	Epithelial/Endothelial	Immune				
Well plates/Cell culture inserts	Caco‐2	–	–	TNF‐α (50; 3–6 h)	Protein expression (p65, p‐IKKα/β, p‐IκBα, COX‐2, and Nrf2); Gene expression (IL‐6, HO‐1, and NQO‐1); Release of TXB_2_ and PGE_2_; TAA^b)^; GSH^c)^	[243]
	Caco‐2	–	–	TNF‐α (10; 48 h)	TEER; P_app_^4)^; LDH; Gene expression (MLCK); Protein expression (claudin‐1 and −4, occludin, ZO‐1, MLC2, phospo‐MLC2, β‐actin, and F‐actin)	[179]
	Caco‐2	–	–	LPS (10^4^; 24 h)	TEER; P_app_; Protein expression (occludin, ZO‐1, claudin‐5, MMP‐9, IκBα, p65, Nrf2, and HO‐1); Gene expression (TNF‐α, IL‐6, COX‐2, and iNOS)	[244]
	Caco‐2	–	–	TNF‐α (50; 6 h)	Gene expression (IL‐8 and IL‐6)	[245]
	Caco‐2	–	–	TNF‐α and IFN‐γ (10) or H_2_O_2_ (1.7 × 10^4^)	TEER; P_app_; ROS; Protein expression (occludin and ZO‐1)	[245]
	Caco‐2	–	–	LPS (5 × 10^4^; 48 h)	Cell viability; Apoptosis; Secretion of TNF‐α	[184]
	Caco‐2	–	–	TNF‐α (50; 6 h)	Activation of NF‐κB and Nrf2 pathways and IKK complex; Gene expression (IL‐8, GCLC, and NQO1); Protein expression (COX‐2); ROS	[182]
	Caco‐2 or HT29	–	–	LPS (100 – 5 × 10^4^; 24–72 h)	Secretion of IL‐8, IL‐6, IL‐1β, TNF‐ α, and IL‐10	[246]
	HT29	–	–	IFN‐γ (50; 12 h) and LPS (10^3^; 15 h)	Secretion of IL‐6, TNF‐α, and IL‐1β; Protein expression (CRP, Claudin‐1, Occludin, TLR4, CD14, and p65)	[247]
	HT29	–	–	TNF‐α (1)	Cell viability; Gene expression (CXCL‐1, IL‐8 and COX‐2, and PPAR‐y); Protein expression (PPAR‐y)	[9]
	Caco‐2; HT29‐MTX	–	–	Short‐term: IL‐1β (10) or hypoxia; Long‐term: IL‐1β (10) or TNF‐α (10) or hypoxia	Protein expression (ZO‐1, MUC2, MUC5AC, ALP, MMP2, MMP9, ADAMTS1, and Caspase 3); Mucus assessment	[186]
	HT29	PBMCs	–	LPS (100; 24 h)	Secretion of TNF‐α; GSH; ROS; Lipid peroxidation	[248]
	Caco‐2; HT29‐MTX	PBMCs	–	PBMCs: OPP (10^3^) + IL‐1β (50) or LPS (100); Epithelial cells: IL‐1β (25) + IFN‐γ (50) + TNF‐α (50) + LPS (10^3^)	TEER; Secretion of PGE_2_, IL‐1β, IL‐6, TNF‐α, and IL‐10; Activity of COX‐1 and COX‐2; Protein expression (COX‐1 and COX‐2)	[249]
	Caco‐2	PBMCs	–	LPS (10^3^) or αCD3 + αCD28 antibodies (1:10 000)	TEER; P_app_; Viability; Secretion of IL‐8, IL‐17a, IFN‐γ, IL‐10, TNF‐α, IL‐1β, and IL‐6; T‐cell phenotype	[250]
	Caco‐2	THP‐1	–	IFN‐γ (10; 24 h Caco‐2 priming) + IFN‐γ + LPS (10; 4 h THP‐1 priming)	TEER; P_app_; Secretion of IL‐1β, IL‐8, TNF‐α, TGF‐β, IL‐4, IL‐6, IFN‐γ, and NO_2_^−^; LDH; Protein expression (F‐actin and ZO‐1)	[95]
	Caco‐2	THP‐1	–	Same as Kämpfer et al., 2017	TEER; Secretion of IL‐1β, IL‐8, TNF‐α, MCP‐1, MIP1α, IFN‐γ, IL‐4, and IL‐6; LDH; Nuclei fragmentation	[185]
	Caco‐2; HT29‐MTX‐E12	THP‐1	–	Same as Kämpfer et al., 2017	TEER; Secretion of IL‐1β, IL‐6, IL‐8, and TNF‐α; Protein expression (ZO‐1); Mucus assessment	[92]
	Caco‐2; HT29‐MTX‐E12	THP‐1	–	Same as Kämpfer et al., 2017	Secretion of IL‐8, TNF‐α and IL‐6; LHD; DNA damage; Protein expression (ZO‐1); Mucus assessment	[94]
	Caco‐2; HT29‐MTX‐E12	THP‐1	–	Same as Kämpfer et al., 2017	TEER; LDH; Secretion of IL‐1β, IL‐6, IL‐8, and TNF‐α; DNA damage	[93]
	Caco‐2	THP‐1	–	Same as Kämpfer et al., 2017	Secretion of IL‐1α, IL‐1β, IL‐6, IL‐8, IL‐10, IL‐10, IL‐12B, IL‐12, IL‐17, and TNF‐α; Gene expression (IL‐8, IL‐1β, IL‐6, TNF‐α, IL‐12A, IL‐23A, MMP9, Muc2, NOS2, TGF‐β1, TFF3, and ZO‐1)	[99]
	Caco‐2; HT29‐MTX	THP‐1	–	Coculture: LPS (5 × 10^4^) or TNF‐α (5, 25 and 100) or IL‐1β (100); Triculture: LPS (10)	TEER; P_app_; Release of IL‐6, IL‐8, TNF‐α, and IL‐1β; Protein expression (ZO‐1 and MUC5AC); Cell viability	[96]
	Caco‐2	THP‐1	–	LPS (10^3^; 3 h)	TEER; Secretion of IL‐1β, IL‐6, IL‐8, and TNF‐α; Gene expression (IL‐1, NF‐κB, and COX‐2)	[98]
	Caco‐2	THP‐1	–	LPS (100)	Secretion of TNF‐α, IL‐1β, IL‐6, and IL‐10; Gene expression (TNF‐α, IL‐1β, IL‐6, and IL‐10)	[97]
	Caco‐2	THP‐1; MUTZ‐3	–	IL‐1β (10; 48 h)	TEER; Protein expression (occludin); Secretion of IL‐8	[188]
	Caco‐2; HT29‐MTX	THP‐1	*E. faecalis, E. coli, S. salivarius, S. mitis, L. plantarum, V. parvula, V. atypica and P. intermedia*	LPS (10; 2 h)	TEER; Cell viability; Secretion of IL‐6, IL‐8, TNF‐α, and CXCL16; Protein expression (Actin); Gene expression (TLR‐2 and ‐4 and DUOX2)	[193]
	Caco‐2; HT29‐MTX	–	*F. prausnitzii, R. intestinalis, B. faecis*	TFN‐α (100) + IL‐1β (25) + INF‐γ (50) for 10 h and LPS (10^4^) for 48 h	TEER; P_app_; Secretion of IL‐8 and MCP‐1; Protein expression (occludin and claudin‐2)	[194]
Organoids	Colonic tissue from IBD and non‐IBD patients	–	–	IL‐1β + IL‐6 + TNF‐α cocktail (10; 4–7 d)	Gene expression (MCP1, IL‐8, IP‐10, Cyclin D1, Ki67, Lgr5, Ephrin B2, KRT20, CA1, and Muc2); Protein expression (ZO‐1, Occludin, Claudin‐1, Ki67, Krt20, CgA, and MUC2); Metabolic activity; Cell viability	[202]
	Colonic tissue from IBD patients	–	–	–	Protein expression (Lgr5, Ki67, L‐FABP, and Muc2); whole genome microarray; gene expression (LYZ, PLA2G2A, ZG16, CLC1, AQP8, MUC12, CLDN18, ANXA10, HYAL1, and SERPINB2)	[203]
	Terminal ileum from CD and non‐CD patients	–	–	–	Protein expression and gene expression (Dsg2, Dsc2, E‐cadherin, and Claudin‐1, ‐2, ‐4, ‐5)	[204]
	Terminal ileum from CD and non‐CD patients	–	–	–	Protein expression (OLFM4, SLC12A2, and E‐Cadherin); microfluidic‐based single‐cell multiplex gene expression (LGR5, OLFM4, ASCL2, BMI1, LRIG1, MSI1, HOPX, MYC, SMOC2, PROM1, SLC12A2, and KCNQ1)	[206]
	Ileum and colon biopsies from IBD and non‐IBD patients (organoid and monolayer)	–	–	IFN‐γ, TNF‐α, IL‐1α	TEER; P_app_; Gene expression (LGR5, MUC2, and ALPI); RNAseq; Histological analysis (H&E, Muc2, and Ki67); Mucus assessment	[207]
	Small intestine from CD and non‐CD patients	–	–	TNF‐α (30)	Organoid‐forming efficiency; Viability assay; Wound healing assay; RNAseq; Protein expression (E‐Cadherin, OLFM4, Lysozyme, CC3, Lgr5, and BMI1); DNA damage; Mucus assessment	[208]
	Embryonic‐derived	–	–	IL‐1α (5) + TNF‐α (50) (12 h)	Gene expression (Fibronectin, Collagen Type I and III, TF, α‐SMA, CXCL10, CXCL1, CCL2, CXCL8 and CCL20, Occludin, Claudin‐1, JAM‐A, and ZO‐1); Secretion of CXCL10, CXCL1, CCL2, CXCL8, and CCL20	[123]
	Small intestine and colonic samples from IBD and non‐IBD	–	–	TNF‐α (100), IFN‐α (50), IFN‐γ (10), IL‐1β (20), IL‐4 (10), IL‐6 (50), IL‐22 (20), IL‐24 (100), IL‐10 (10), IL‐12 (20), IL‐17A (5 × 10^7^), IL‐33 (10) and TL1A (100) (30 min)	Protein expression (E‐Cadherin, pan‐cytokeratin, Musashi‐1, and Ki67); Organoid swelling; Gene expression and secretion of CFTR, NKCC1, KCNQ1, and KCNN4	[251]
	iPSC‐derived (monolayer)	–	–	IL‐17 (0.04‐25), IFN‐γ (0.01‐1), IL‐22 (1‐10) (2 days)	TEER; Protein expression (villin1, E‐Cadherin, Lysozyme, Muc2, and ChgA); Gene expression (villin1, pIgR and Reg3β, MMP9, IL‐6, and TNF)	[34]
	iPSC‐derived	–	–	TNF‐α (30) (96 h); TNF‐α (30) + TGF‐β (2) (48 h)	Protein expression (villin, Lgr5, CDX2, Muc2, E‐Cadherin, ChgA, Lysozyme, ZO‐1, Occludin, Vimentin, and α‐SMA); Gene expression of intestinal epithelial cells markers, mesenchymal markers and inflammatory cytokines; P_app_	[210]
	iPSC‐derived	–	–	IFN‐γ (1‐1000) (3 days)	Gene expression (IDO1, CXCL10, GBP1, GBP5, TRIM22, and WARS); Protein expression (E‐Cadherin, ZO‐1, and p‐STAT1); Transcriptional profiling by microarray;	[211]
	iPSC‐derived	Monocytes from healthy buffy coats	Heat‐inactivated *A. fumigatus*	LPS (10^4^), poly(I:C) (2 × 10^4^), BGP (5 × 10^3^), ODN2216 (5 × 10^3^), Zymosan (5 × 10^3^) (24 h)	Protein expression (E‐Cadherin, CDX2, ASCL2, and CD45); Gene expression (CDX2, ASCL2, TLR2, TL3, TLR4, TLR7, TLR9, IL‐1β, IL‐6, IL‐8, IL‐12p40, MCP‐1, TNF‐α, COX‐2, CRP, MMP‐9, S100A8/9, and β‐defensin‐2, −5); Secretion of CRP and S100A8/9; Cytometric bead immunoassay (IL‐1β, IL‐6, IL‐8, IL‐10, IL‐12p70, IL‐17A, IL‐18, IL‐23, IL‐33, IFN‐α, IFN‐γ, MCP‐1, and TNF‐α); NF‐κB quantification	[137]
	iPSC‐derived	PMNs^e)^	*E. coli* (commensal and pathogenic)	LPS (apical or basolateral – 0.01–10 ng, 120 h)	P_app_; ROS production; Protein expression (E‐Cadherin and F‐actin); Gene expression of epithelial cell and mesenchyme markers, and inflammatory cytokines; PMN migration	[138]
	Ileal samples‐derived (monolayer)	PMNs	*S. flexneri*		TEER; Secretion of MCP‐1, TNF‐α, IL‐1β, IL‐6, and IL‐8; PMN migration; Protein expression (CD45 and F‐actin)	[139]
	Colonic samples from IBD and non‐IBD	Lamina propria leukocytes and BM^f)^ dendritic cells	–	–	Protein expression (Hes1 and UEA‐1); Gene expression (Lgr5, Muc2, ChgA, E‐Cadherin, and Hes1);	[141]
	Duodenal and jejunal samples‐derived (monolayer)	PBMCs	*E. coli* (toxigenic and pathogenic)	–	TEER; Bacterial killing assay; Protein expression (Muc2, Lysozyme, Phospho‐Ezrin, Chromogranin‐A, and CD14); Secretion of IL‐8, IL‐1β, IL‐2, IL‐4, IL‐10, IL‐13, IL‐12p70, TNF‐α, IFN‐γ, IL‐6, and TGF‐β1	[212]
	iPSC‐derived	Lamina propria mononuclear ILCs^g)^ from mouse and IBD patients	–	–	Protein expression (CD45, ZO‐1, CD44, E‐Cadherin, F‐actin, β‐catenin, SMA, Vimentin, and TGF‐βR); Gene expression (INF‐γ, IL‐22, and TGF‐β1); Secretion of TGF‐β1	[131]
	Colonic tissue (monolayer)	Monocyte‐derived Macrophages from whole blood	–	LPS (100) + INF‐γ (20)	SEM; Protein expression (E‐Cadherin, Muc2, CD68, and iNOS); macrophage infiltration; Mucus assessment; Inflammatory cytokine array	[136]
Gut‐on‐chip	Caco‐2; Human endothelial cells	PBMCs	VSL#3: 8 probiotic microbes	Enteroinvasive *E.coli*; LPS (1.5 × 10^4^); L‐1β (3), IL‐6 (5), IL‐8 (15), and TNF‐α (4)	TEER; Villi height; Protein expression (ICAM‐1, IL‐1β, IL‐6, IL‐8, TLR4, and TNF‐α); Transcriptomics (IL‐1α, IL‐1β, IL‐2, IL‐4, IL‐6, IL‐8, IL‐10, IL‐12, IL‐17A, IFN‐ γ, TNF‐α, and GM‐SCF)	[222]
	Caco‐2; HUVECs	‐	*L. plantarum* (HY7715); *B. animalis* spp. Lactis (HY8002)	LPS (1.5 × 10^4^; 24 h)	TEER; Protein expression (ZO‐1)	[252]
	Caco‐2	PBMCs	VSL#3: 8 probiotic microbes	LPS, *E*. coli; DSS	TEER; Villi height; P_app_; Live/dead; LDH; ROS; Protein expression (ZO‐1, E‐cadherin, IL‐1β, IL‐6, and TNF‐α); Mucus assessment	[253]
	Caco‐2	–	*L. rhamnosus GG*	High‐fibre dietary regimen	Gene expression (qRT‐PCR of ABCC3, ABCA5, ABCC2, COX‐2, TRAF6, and c‐JUN); RNAse	[231]
	Caco‐2	–	–	IL‐1β (2), IFN‐γ (100) TNF‐α (100)	TEER; Protein expression (MIP3α, IP‐10, IL‐8, IL‐6, IL‐1β, TNF‐α, and E‐cadherin)	[254]
	Caco‐2	THP‐1; Neutrophils	–	LPS; fLMP	P_app_; Protein expression (IL‐1β, MIP1α, MIP1β, TNF‐α, IL‐6, IL‐8, and MMPs); LDH; Neutrophil influx	[233]
	Caco‐2; HT29‐MTX	THP‐1; MUTZ‐3	–	TNF‐α (200‐300); IL‐1β (200)	TEER; P_app_; Protein expression (IL‐8)	[255]
	iPSCs	–	–	TNF‐α, IL‐1β and IFN‐γ (50)	Protein expression (IL‐6 and IL‐8); Gene expression (MIP3α, IL‐6, and IL‐8)	[126]
	Caco‐2; HUVECs	PBMCs	*L. rhamnosus*	LPS	P_app_; Protein expression (TNF‐α, IL‐1β, IL‐6, IL‐8 and IL‐10, CD68, CD103, and CX3CR1)	[256]
	Colon‐derived organoids; HUVECs; primary fibroblasts	THP‐1	–	TNF‐α	Monocyte infiltration; Protein expression (ICAM‐1)	[257]
	Organoid‐derived intestinal epithelium; HIMECs	PBMCs	–	OC34 or NL63 (coronaviruses)	P_app_; Protein expression (VE‐cadherin and Caspase 3); PBMC recruitment; Secretion of IL‐8, IL‐6, MCP‐1, MIP1α, IP‐10, IL‐33, IFN‐γ, and NGAL	[230]
	Caco‐2	–	–	LPS (10^5^; 24 h)	TEER; P_app_; Protein expression (IL‐8)	[258]

^a)^ ng mL^−1^;

^b)^ TAA – Intracellular total antioxidant activity;

^c)^ GSH – Reduced glutathione;

^d)^ P_app_ – Permeability;

^e)^ PMNs – Polymorphonuclear leukocytes;

^f)^ BM – Bone Marrow;

^g)^ ILC – Innate Lymphoid Cell.

### 5.1 Multiwell Plate and Cell Culture Insert‐Based Models

Early in vitro models of gut inflammation were simplistic and normally based on single cultures on a dish or microplate. Later, the use of cell culture inserts provided straightforward solutions for in vitro studies, allowing the evaluation of barrier function and intestinal permeability by granting access to both apical and basolateral sides of polarized cell monolayers. Data obtained from these simple approaches revolutionized some physiological concepts and paved the way for the development of more sophisticated models.^[^ ^177^ ^]^

Despite the simplicity of monocultures, lacking many features of the human intestine, they remain useful to study the response of different cell types to inflammatory stimuli, preceding their use in more advanced settings. Van de Walle et al.^[^ ^178^ ^]^ used Caco‐2 cells to study how different stimuli concentrations and combinations impacted inflammation. IL‐1β and TNF‐α led to IL‐8 secretion, while LPS and IFN‐γ showed no effect. However, when applied together there was maximal production of IL‐8 and IL‐6. IL‐1β also increased the levels of prostaglandin E2 (PGE2). The formation of nitric oxide (NO) seemed to depend on the presence of IFN‐γ, and TEER only decreased when the authors added IL‐1β to the basolateral compartment. Watari and colleagues^[^ ^179^ ^]^ exposed Caco‐2 cells to TNF‐α and observed an upregulation of MLCK genes, and consequent MLC phosphorylation and TJ disruption.^[^ ^87^, ^180^, ^181^ ^]^ This contrasted with the work by Van de Walle and colleagues, where stimulation with TNF‐α did not affect TEER. Speciale et al.^[^ ^182^ ^]^ also used TNF‐α to induce inflammation in Caco‐2s and observed the activation of the NF‐κB pathway, overexpression of IL‐8, and increased production of reactive oxygen species (ROS). In another study, Caco‐2 cells were stimulated with LPS, which decreased the TEER after 24 h, again contradicting the findings of Van de Walle and colleagues.^[^ ^183^ ^]^ In this study, LPS increased the expression of IL‐6, INOS, TNF, and cyclooxygenase‐2 (COX‐2) genes and the production of iNOS and COX‐2 at the protein level. Another study that used LPS in Caco‐2 monolayers observed a marked decrease in cell viability and an increase in apoptotic‐like cells and TNF‐α secretion.^[^ ^184^ ^]^

Using cocultures increases the physiological relevance of the models, and the inclusion of immune cells is key to mimic inflammation. Kordulewska et al.^[^ ^96^ ^]^ used cocultures of Caco‐2 and THP‐1 stimulated with LPS, and found that LPS promoted a decrease in TEER of about 40%, while promoting Caco‐2 proliferation. La Paz and colleagues,^[^ ^97^ ^]^ in their turn, showed an increased production of ROS, nitrites, and pro‐inflammatory cytokines. There was a tenfold difference in the concentration of LPS used in the studies and interestingly when higher concentrations were used, lower levels of pro‐inflammatory cytokines were detected. Kämpfer and co‐workers^[^ ^95^ ^]^ cocultured Caco‐2 with macrophage‐differentiated THP‐1 cells to establish both healthy and inflamed intestinal epithelium models. In the healthy condition, THP‐1 led to a temporary reduction in TEER (10%) that was re‐established within 24 h. In the inflamed condition, TEER was reduced by 20–25% for 24 h, along with a significant increase in cytokine secretion. The authors induced inflammation by priming Caco‐2 cells with IFN‐γ for 24 h and coculturing them with the macrophage‐differentiated THP‐1 cells, which were primed with IFN‐γ and LPS for 4 h. The authors reported an increase of nitrite and LDH activity, and of IL‐1β, TNF‐α, IL‐8, and other cytokines when compared to the healthy model, but found similar paracellular permeability.^[^ ^95^ ^]^ Kämpfer et al.^[^ ^185^ ^]^ used the same model to investigate the impact of inflammation at the time of exposure to nanomaterials and observed clear differences in the sensitivity of Caco‐2, which were more sensitive under inflammatory conditions. Curiously, in a following work where HT29‐MTX cells were added to the model, ongoing inflammation did not affect the inflammatory response.^[^ ^94^ ^]^ Marescotti and co‐workers^[^ ^98^ ^]^ also established a model of the inflamed mucosa combining Caco‐2 and HT29‐MTX with THP‐1‐differentiated macrophages and observed a decrease in TEER and an increase in intestinal permeability and cytokine release. The authors claimed the model was suitable to assess the anti‐inflammatory properties of compounds after pre‐treating the cells with budesonide and TPCA‐1 and confirming the prevention of LPS‐induced inflammation.

3D models provide improved and more physiological support aiming to better mimic the intestinal environment. Dosh and colleagues^[^ ^186^ ^]^ established a 3D model to mimic the healthy and diseased intestine for an extended period of time. The authors used Caco‐2/HT29‐MTX cocultures layered on l‐pNIPAM and established short and long‐term cultures of 6 and 11 weeks respectively. The cultures were stimulated with IL‐1β and TNF‐α or subjected to hypoxia for 1 week. The authors concluded that the l‐pNIPAM hydrogel and dynamic culture conditions (gentle shaking) enabled long‐term culture and allowed to investigate cytokine effects on cell behavior. Leonard and colleagues^[^ ^187^ ^]^ established a 3D intestinal model by embedding PBMC‐derived monocytes and DCs in collagen and seeding Caco‐2 cells on top. They observed that when challenged with IL‐1β, the model responded with an increased inflammatory cytokine response when compared to a Caco‐2 monoculture. The same group later used THP‐1 and MUTZ‐3 cell lines as surrogates for macrophages and DCs respectively, to circumvent the variability associated with primary cells. The models were also challenged with IL1‐β and the inflamed cocultures showed a decrease in TEER, an increase in IL‐8 secretion, and disruption of TJs.^[^ ^188^ ^]^

**Figure 4:**
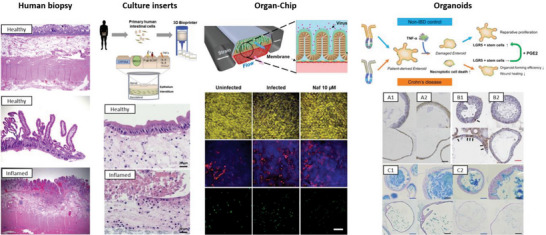
Different set‐ups and devices used in in vitro models of intestinal inflammation. Histological sections of human biopsy tissue stained with H&E show a normal histology of the small bowel (top) and of the small intestinal mucosa (middle). The bottom image shows a section of the small bowel with early involvement by Crohn's disease. Reproduced with permission,^[^ ^240^ ^]^ Copyright 2018, Academic Press. The approach in culture inserts shows the work by Madden et al.^[^ ^189^ ^]^ where bioprinting was used to achieve a multi‐layered tissue incorporating intestinal myofibroblasts and human intestinal epithelial cells. An inflamed state was achieved via stimulation with TNF‐α. Reproduced with permission.^[^ ^189^ ^]^ Copyright 2018, Cell Press. The Organ‐chip example depicts the work by Bein et al.^[^ ^230^ ^]^ where an intestine‐on‐a‐chip lined by patient organoid‐derived intestinal epithelium was used to study inflammatory responses to infection with a coronavirus and resolution following treatment with a protease inhibitor drug – nafamostat (Naf). Reproduced with permission,^[^ ^230^ ^]^ Copyright 2021, Frontiers. Finally, the Organoids example shows patient‐derived enteroids (non‐IBD controls and Crohn's) stimulated with TNF‐α to obtain inflamed enteroids and assess the effect of PGE2. A1 to C2 show immunohistochemistry of the control (1) and diseased (2) enteroids for (A) E‐cadherin and (B) LGR5. C1 and C2 show Alcian blue staining of the control (C1) and diseased (C2) enteroids. Reproduced with permission,^[^ ^208^ ^]^ Copyright 2022, Elsevier.

Primary cells, although not as frequently, are also used in insert‐based models. Madden et al.^[^ ^189^ ^]^ bioprinted a 3D intestinal tissue using human epithelial cells from donors and commercially available myofibroblasts (**Figure** **4**). The tissues included subpopulations of goblet cells, Paneth cells, and enteroendocrine cells, expressed relevant enzymes (CYP3A4) and transporters (P‐gp and BCRP), and presented a stable barrier and selective permeability to different markers. Besides, they were responsive to inflammatory stimuli: challenging the tissues with TNF‐α resulted in cell death and upregulation of inflammatory cytokines (Figure 4). Another study used primary stem cells from the small and large intestine and established monolayers with stable TEER, presence of ALP^+^ colonocytes and Muc2^+^ goblet cells, and spontaneous secretion of the pro‐inflammatory cytokines IL‐8 and MCP‐1.^[^ ^120^ ^]^ The authors also induced inflammation with TNF‐α and studied the potential anti‐inflammatory effects of dietary compounds. Wang and colleagues (Wang et al., 2020) induced inflammation on the MatTek EpiIntestinal model by challenging the tissue with TNF‐α and IFN‐γ. Simon and co‐workers^[^ ^118^ ^]^ used the same MatTek model to show that exposure to IL‐6 decreased the transcription of CYP3A4 mRNAs.

The work by Madden et al. was one of the earliest examples of a model of human inflammation obtained by bioprinting.^[^ ^189^ ^]^ Although examples of bioprinted intestinal models tackling inflammation are still lacking, it is clear that this fabrication strategy will play an important role in the development of models with high complexity owing to its capacity to combine different cell types and biological materials with high special resolution. This is well exemplified by the recent work by Torras et al. where digital light projection stereolithography was used to fabricate complex crypt‐villus structures laden with fibroblasts on top of which Caco‐2 were later seeded.^[^ ^190^ ^]^ The authors also showed that a 3D architecture impacts significantly the expression of relevant drug transporters, which can affect the management of IBD using anti‐inflammatory drugs.^[^ ^191^ ^]^ Also recently, Cheng et al. bioprinted a channel in GelMA lined with Caco‐2 to study the inflammatory pathways triggered by cocultures with *Salmonella enterica* and *Lactobacillus reuteri* in both aerobic and anaerobic conditions. However, the cocultures were only kept for 12 h.^[^ ^192^ ^]^

Microbiota are normally absent in static cell‐based in vitro models due to bacterial overgrowth, which makes long‐term cultures hard to attain.^[^ ^108^ ^]^ Calatayud et al.^[^ ^193^ ^]^ developed a model with Caco‐2/HT29‐MTX, THP‐1‐differentiated macrophages, and eight strains of commensal bacteria. The authors observed that adding microbiota did not affect the TEER, but slightly increased the permeability of Lucifer Yellow (LY) and increased TNF‐α, IL‐8, and CXCL16 production. Mohebali and colleagues^[^ ^194^ ^]^ added the intestinal commensal strains *F. prausnitzii*, *R. intestinalis*, and *B. faecis* alone or in combination to inflamed Caco‐2 and HT29‐MTX cells and observed an anti‐inflammatory effect: all strains increased TEER, decreased paracellular permeability, and decreased IL‐8 and MCP‐1 release. To avoid bacterial overgrowth, in both studies the microbiota were cocultured with cells for short periods of time (2 and 6 h). Organ‐chips arise as a promising alternative since they enable the use of continuous flow, oxygen gradients, and mechanical deformation, avoiding bacterial overgrowth while maintaining cellular viability.^[^ ^108^ ^]^

### 5.2 Organoids

Organoids are multicellular 3D structures that can recapitulate the complex cellular organization found in vivo, overcoming reductionist in vitro models.^[^ ^195^ ^]^ Intestinal organoids or “mini‐guts”, as often called, emerged in the last decade as a promising alternative to model inflammatory scenarios and to explore the role of the epithelium and its surrounding environment upon mucosal injury.^[^ ^167^, ^196^, ^197^, ^198^ ^]^

Organoids are frequently derived from adult stem cells, isolated from the small intestine or the colonic epithelium, giving rise to sophisticated constructs termed enteroids or colonoids, respectively.^[^ ^199^ ^]^ To culture in vitro, isolated cells are typically embedded in an extracellular matrix‐rich hydrogel, and indefinitely propagated using a growth‐factor defined environment to generate near‐native epithelial cell clusters.^[^ ^200^ ^]^ These clusters are physiologically more relevant than standardized cell lines, for they contain all epithelial cell types and can self‐organize into hierarchical, polarized and lumenized structures, with crypt‐villi domains. These assemblies also perform basic functions such as molecular absorption, transport, and endocrine and paracrine secretion, highly resembling the in vivo environment.^[^ ^198^, ^201^ ^]^

The generation of IBD patient‐derived organoids constitutes an interesting approach from a personalized medicine standpoint, since cells maintain an authentic epigenetic background of the disease, resulting from chronic exposure and impregnation of inflammatory mediators.^[^ ^202^, ^203^, ^204^, ^205^, ^206^ ^]^ Organoids derived from IBD‐afflicted patients revealed a set of differentially regulated genes associated with antimicrobial defense, secretory, and absorptive functions when compared with their non‐IBD counterparts. Such differences in expression profiles show that permanent changes in the epithelium of affected individuals can be promoted by alterations imprinted in the stem cell compartment, which may contribute to the perpetuation of disease.^[^ ^203^ ^]^ d'Aldebert and colleagues^[^ ^202^ ^]^ demonstrated that colonoids from IBD patients without any further immunological stimulation presented a classical inflammatory phenotype, with an abnormal epithelium polarization and impaired barrier function, decreased expression of tight junction proteins and inferior budding capacity. However, Noben and co‐workers^[^ ^205^ ^]^ highlighted that inflammatory cues at the mRNA level may not be propagated from biopsy tissues to organoid cultures, and that additional stimuli might be necessary to extend the expression of other markers. Supporting this idea, another study revealed that only enteroids generated from sites of severe inflammation conserved the changes in junctional proteins when compared to the ones generated from sites with mild inflammation.^[^ ^204^ ^]^ In contrast, other studies reported having difficulties in generating long‐term cultures with organoids from active inflamed regions.^[^ ^207^, ^208^ ^]^

Despite being an appealing tool to study inflammatory mechanisms, organoids still have roadblocks to surpass.^[^ ^169^ ^]^ Generally, Lgr5^+^ adult stem cell‐derived organoids only comprise the epithelia, lacking a stroma or lamina propria.^[^ ^198^, ^209^ ^]^ To recapitulate the inflammatory phenotype and its typical outcomes using organoid cultures, two different approaches are described: (i) delivery of pro‐inflammatory mediators in the absence of immune cells,^[^ ^123^, ^202^, ^210^, ^211^ ^]^ and (ii) coculture with different immune cell subpopulations.^[^ ^131^, ^136^, ^137^, ^138^, ^139^, ^141^, ^212^ ^]^ In the first strategy, exposure to immune cell‐derived pro‐inflammatory cytokines and immunostimulatory microbial components influences epithelial cell survival and behavior.^[^ ^34^ ^]^ Interestingly, Lee and team^[^ ^208^ ^]^ demonstrated that TNF‐α promotes necroptosis of intestinal epithelial cells, especially affecting differentiated cell populations, while inducing intestinal stem cell expansion (Figure 4). When the damage is mild, not completely eradicating the stem cell pool, or limited to the differentiated compartment, TNF‐α promotes the survival of different subsets of stem cells in both control and CD‐derived enteroids. Another work shows the decreased expression of TJ proteins when “healthy” colonoids are exposed to an inflammatory cocktail, producing a similar response to the one seen in IBD‐derived organoids.^[^ ^202^ ^]^ Furthermore, the latest research using pluripotent stem cell‐derived organoids showed the effects of inflammatory stimuli on the expression of mesenchymal and fibrotic markers – intestinal fibrosis is another hallmark of chronic IBD beyond the characteristic epithelial damage.^[^ ^123^, ^210^ ^]^ The use of pro‐inflammatory molecules focuses primarily on the effects exerted on epithelial cells, misrepresenting the complexity found in the native environment and the intricacy of epithelial‐immune cell crosstalk. This bidirectional relationship can be studied by coculturing immune cells with the epithelium. In this perspective, and due to performing distinct and specific functions in inflammation, different subsets of immune cells have been used in combination with organoids models: monocytes,^[^ ^137^ ^]^ macrophages,^[^ ^136^, ^212^ ^]^ polymorphonuclear leukocytes,^[^ ^138^, ^139^ ^]^ lymphocytes,^[^ ^130^ ^]^ and DCs.^[^ ^141^ ^]^

Another drawback of organoids lies in their heterogeneous structures with a closed sphere‐like spatial arrangement that prevents direct access to the apical side of the epithelium.^[^ ^213^ ^]^ Access is only possible by resourcing to technically challenging methods, such as microinjection^[^ ^214^ ^]^ or polarity inversion,^[^ ^215^ ^]^ rendering organoids unsuitable for standard functional assays designed for flat monolayers – such as permeability studies or TEER measurements.^[^ ^216^ ^]^ Organoid‐derived monolayers exploited in conventional 2D formats, such as cell culture inserts, or even in microfluidic systems are a compelling and convenient option to overcome such challenges, allowing manipulation of both apical and basolateral sides in a compartmentalized manner. In addition, the combination of organoid technologies with organ‐on‐chip platforms allows the development of more complex models including dynamic and physiological features, such as the recreation of intestinal motility and luminal flow, mechanical strain, and shear stress.^[^ ^217^, ^218^ ^]^ However, organoid cultures and their derived monolayers exhibit different transcriptomic profiles, suggesting that gene expression is governed by cellular organization and architecture and thus such technology combinations should be pursued with care.^[^ ^207^ ^]^

### 5.3 Organ‐on‐Chip

Organ‐on‐chip uses microfabricated channels or structures to host living cells or tissues to replicate the functionality of living organs. The first reports of organ‐on‐chip devices mimicking the gut date back to the late 2000s,^[^ ^219^ ^]^ and multiple companies, such as Emulate Inc., MIMETAS, and others, are now commercializing such devices. Organ‐chips are attractive for allowing precise tuning of dynamic fluid flows, high spatiotemporal resolution of nutrient/stimuli delivery^[^ ^220^ ^]^ and outflow sampling,^[^ ^221^ ^]^ the introduction of mechanical cues such as peristalsis,^[^ ^222^ ^]^ and online real‐time sensing of multiple parameters, including O_2_ concentration,^[^ ^220^, ^223^, ^224^ ^]^ pH,^[^ ^223^ ^]^ or trans‐epithelial electrical resistance.^[^ ^225^, ^226^ ^]^ As stated previously, fluid flow is key to enable long‐term cocultures with gut microbiota through the continuous delivery of nutrients, and the removal of waste products and exceeding bacterial growth. The critical role of the gut microbiota on intestinal homeostasis and inflammation positions organ‐chips in the front row as state‐of‐the‐art in vitro gut inflammation models.

In 2015, Kim et al.^[^ ^227^ ^]^ used the Gut Chip now commercialized by Emulate Inc., to replicate the intestinal epithelium using Caco‐2 and microvascular/lymphatic endothelial cells grown on opposite sides of a semiporous membrane separating two overlapping channels. The introduction of a pathogenic strain of enteroinvasive *E. coli* elicited a sharp reduction in TEER. Interestingly the barrier integrity was maintained when the epithelium was challenged with LPS from pathogenic *E. coli*. But when LPS was used together with human PBMCs, not only the TEER was drastically reduced but there was also significant production of pro‐inflammatory cytokines (IL‐1β, IL‐6, IL‐8, and TNF‐α), increased expression of intercellular adhesion molecule (ICAM)−1 by the endothelial cells, and upregulation of Caco‐2 genes encoding proteins that mediate early inflammatory signaling and leukocyte recruitment. A later study with the same device used dextran sodium sulphate (DSS) to impair epithelial barrier integrity, villi microarchitecture, and mucus production.^[^ ^228^ ^]^ The sensitization of the epithelium showed that a “leaky gut” can make the epithelium more prone to oxidative stress and subsequent inflammatory cascades mediated by intercellular host–microbiome crosstalk. Another study with gut microbiota showed that the coculture of a damaged epithelial barrier (inflamed using LPS) with probiotics (HY7715) sped up the recovery of barrier function.^[^ ^229^ ^]^ Bein and colleagues^[^ ^230^ ^]^ used organoid‐derived intestinal epithelium and vascular endothelium in the Intestine‐Chip from Emulate to study cellular and inflammatory responses to infection with a coronavirus (Figure 4). The viral infection decreased the junctional protein VE‐cadherin and increased the apoptotic marker Caspase 3. It also decreased barrier function and increased cytokine production and recruitment of PBMCs.

Greenhalgh and colleagues^[^ ^231^ ^]^ cocultured Caco‐2 cells with *L. rhamnosus* GG in the HuMiX device to evaluate pro‐inflammatory pathways associated with exposure to a high‐fiber diet. The study showed an up‐regulation of the IL‐1 signaling pathway, including IL‐1R1, and its downstream targets – TRAF6, COX‐2, and C‐JUN – when the dietary fiber was provided to the cells alone. In contrast, when the prebiotic was administered in symbiosis with *L. rhamnosus* GG, there was a down‐regulation of inflammation‐driven colorectal cancer‐associated signaling pathways, emphasizing the importance of including microbiota in gut inflammation studies.

The OrganoPlate from MIMETAS was used to test whether viral transduction of shRNAs against MYD88 and RELA, a downstream adaptor protein of the toll‐like receptor (TLR) and IL‐1 signaling pathways and a subunit of NF‐κB, respectively, could reduce the inflammation state of Caco‐2 cells following a stimulus with a cocktail containing IL‐1β, TNF‐α, and IFN‐γ.^[^ ^232^ ^]^ The inflammation model was first validated by verifying a reduction in TEER, an increased production of IP‐10, IL‐8, and CCL‐20, and a reduced localization of E‐cadherin at cellular junctions. The study indicated that the *MYD88* and *RELA* knockdowns could partially mitigate the pro‐inflammatory cytokine production triggered in Caco‐2 cells. The same model was later used to study neutrophil infiltration from an adjacent channel when the intestinal epithelium was cocultured with THP‐1‐differentiated macrophages.^[^ ^233^ ^]^ When THP‐1 were stimulated with a combination of LPS and fMLP, there was a significant increase in the production of IL‐1β, Mip1α, and Mip1β, TNF‐α, and IL‐6 in the basolateral channel, and of IL‐6 and IL‐8 in the luminal channel (secreted by Caco‐2). The presence of activated macrophages increased the influx of neutrophils, recapitulating the crosstalk between different cell types that aggravates IBD in vivo. Other studies using the OrganoPlate included Caco‐2 and HT29‐MTX cocultures in combination with differentiated THP‐1 and MUTZ3 to mimic the role of DCs,^[^ ^234^ ^]^ and iPSC‐derived tubular structures including enterocytes, Paneth cells and neuroendocrine cells.^[^ ^235^ ^]^

The MOTiF biochip from microfluidic ChipShop GmbH cultured Caco‐2 and HUVEC cells lining the two sides of a permeable membrane.^[^ ^236^ ^]^ Monocytes from human PBMCs were differentiated into mucosal macrophage and DC phenotypes and cultured on the vascular and luminal side, respectively. When LPS was present in the vascular side, there was release of TNF, IL‐1β, IL‐6, and IL‐8, validating the functionality of the model. The authors further showed that colonization of the model with the probiotic *L. rhamnosus* avoided the overgrowth and translocation of the opportunistic pathogen *C. albicans*.

The IFlowPlate allows the culture of 128 independently perfused and vascularized colon organoids.^[^ ^237^ ^]^ The authors showed that perfusion improved the growth of the organoids when comparing to conventional static conditions. In addition, circulating THP‐1 cells could extravasate from the vasculature in response to TNF‐α stimulus.

Finally, multiorgan‐chip platforms allow for comprehensive inflammation studies that consider the interplay between different organs. Jeon et al.^[^ ^238^ ^]^ and Kim et al.^[^ ^239^ ^]^ used gut‐liver and gut‐brain axis platforms, respectively, to study inflammation pathways triggered by LPS. The former demonstrated the anti‐inflammatory effect of luteolin (reflected by a reduced production of nitric oxide by RAW264.7 cells) following absorption by the gut epithelium and metabolism in the liver (HepG2 cells). The latter showed that butyrate improved the barrier integrity of both Caco‐2 and brain microvascular endothelial cells following administration in the intestinal lumen.

## 6 Main Considerations and Future Perspectives

The small intestine is a complex organ, with multiple layers and a panoply of different cell types and populations that interact in a comprehensive crosstalk that shapes their behavior. During inflammation, the complexity of this macroenvironment increases with the initiation of inflammatory cascades and consequent secretion of numerous compounds. To believe that one will be able to fully mimic this intricate scenario in vitro is unrealistic. Yet, in vitro models are becoming increasingly more accurate and consequently more useful.

The latest achievements in the field have been remarkable and the ability to reproduce unique features of the small intestine and its inflammatory state continues to improve. Throughout this review we showed that many in vitro models can replicate important features of IBD, contributing to the understanding of the disease. Simplistic in vitro models are still widely used and we foresee that this is not yet to change, since they offer a straightforward, reproducible, and cost‐effective means to study the response of cells to specific stimuli. We believe it is important to first comprehend how different cells react to each stimulus before progressing to more complex settings or incorporating multiple stimuli. This knowledge can be crucial both to unravel disease mechanisms and to study unique cellular responses when testing novel compounds. It is our view that these models will continue to be used to obtain preliminary data before proceeding to more complex set‐ups. However, it is equally important to acknowledge that simplistic models may not fully replicate the in vivo environment, potentially resulting in divergent cellular responses from those observed in reality. Moreover, this review also highlighted that studies using similar cell lines and stimuli can lead to different outcomes. While these cells allow high intralaboratory reproducibility, they frequently present inter‐laboratory variability, which impairs result comparisons. This lack of standardization constitutes a huge challenge when trying to integrate such models in the roadmap of food and drug development.

Looking at Table 1, it is clear that simpler models such as well plates and cell culture inserts use the Caco‐2 and HT29‐MTX cell lines most frequently. These cells also find application in gut‐chip devices, although the emergence of induced pluripotent stem cells (iPSCs) and organoid‐derived cells brings them into focus as more physiologically relevant options. Incorporating immune cells is crucial owing to the pivotal role of the immune system in inflammatory disease. Among available immune cell lines, THP‐1 cells are the most widely employed followed by MUTZ‐3. Incorporating diverse primary immune cells such as monocytes, leukocytes, macrophages, and dendritic cells, makes the models more relevant.

Regarding inflammatory stimuli, some cytokines clearly dominate in terms of usage in vitro. TNF‐α, IFN‐γ, IL‐1β, IL‐6, and, in the case of organoids, IL‐1α, are more frequently used due to their pro‐inflammatory profile and their association to IBD. LPS is also extensively employed to induce inflammation. However, there are significant variations regarding the concentrations of the compounds used and the duration of the stimuli. This variability can be attributed to the reality that immortalized cells tend to exhibit different phenotypes across different laboratories, requiring constant optimization of inflammatory stimulus conditions to elicit the desired response. Given the inherent variability, we anticipate that countering this tendency in studies inducing inflammation will prove challenging.

It is also clear that there is no consensus on the number and nature of the measured outputs in intestinal inflammation models. The most commonly assessed parameters include TEER, apparent permeability, viability, secretion of relevant cytokines (which also vary), and the expression of genomic or proteomic markers. Studies utilizing organoids tend to make more comprehensive genetic and protein analyses. The need for a thorough characterization of the organoids arises from their relatively less established nature when compared to immortalized cell lines. However, we believe that a detailed characterization, both in terms of genetic expression and protein profiles, is critical in all models to understand how inflammation shapes genetic information and the extent to which these modifications are translated into proteins.

One frequent pitfall in many studies is the lack of verification regarding the establishment of a mucous barrier, which role in inflammation should not be undermined. The difficulty of precisely assessing mucus in laboratory settings may contribute to this phenomenon. Cell infiltration and migration, which are crucial for understanding the functionality of immune cells during inflammation, are also typically not assessed in most studies; and the few studies that evaluate this, use gut‐on‐chip or organoid models. In our view, another essential aspect of characterization that often lacks in in vitro models mimicking intestinal inflammation is the inclusion of standard anti‐inflammatory compounds to demonstrate the anti‐inflammatory mechanisms of the models.

We trust that organoids will have a starring role in the upcoming years. By being able to mimic organ physiology and to integrate the most important cell types as well as and the crypt‐villi domains, they will become a vital tool to help dissect key inflammation events occurring in vivo, particularly when using cells derived from IBD patients. However, the drawbacks associated with organoid technology, such as the lack of a stromal compartment, immune cells, and microbiota, and the difficult access to the lumen may avert some specific studies. Thus, it is likely that research will focus on the use of organoid cultures combined with other technologies like cell culture inserts, bioprinting, or organ‐chips. While the former is simpler, the latter enables further integration of dynamic intestinal features, such as fluid flow and peristalsis. All offer the potential to use cells derived from organoid cultures while integrating key missing elements: a stromal compartment, immune cells, and the microbiota. Although the integration of microbiota on static cell culture inserts is difficult due to the risk of bacterial overgrowth, thus can potentially be overcome by the integration of set‐ups that allow media circulation, like bioreactors or the MIVO platform, which can add millifluidic flow to static tissue models (Costa et al., 2020).^[^ ^241^ ^]^

Despite being a very promising approach to tissue and disease modelling, only a few studies have exploited the potential of biofabrication to recreate intestinal inflammation. Organ‐ and tissue‐level biological functions are driven by tissue composition, morphology and architecture. To achieve such complexity in vitro, a very recent and relevant study combined organoids and 3D bioprinting to engineer living‐tissue constructs with key hepatic functions.^[^ ^242^ ^]^ The use of this bioprinting technique allowed for a precise control over tissue architectural features, while the use of organoids offered organ‐like morphogenesis and organization with multiple tissue‐specific cell lineages. This approach could be easily translated to other tissues and organs, such as the intestine, and could also be of interest in recreating diseased tissue. In a more challenging and ambitious approach, this could also be incorporated within organ‐chip platforms leveraging the multiple benefits of the three technologies. We believe that the combination of such strategies will open new opportunities to produce models with high‐level biological functions and improved physiological relevance.

## Conflict of Interest

The authors declare no conflict of interest.

## Author Contributions

Study conception and design: M.H.M., M.N., C.G., and M.X.; Article drafting: M.H.M., M.N., C.G., and M.X. Article revision: M.H.M., M.N., L.P., C.G., and M.X. All authors revised and approved the final submitted version.
